# Improvement of Legionnaires’ disease diagnosis using real-time PCR assay: a retrospective analysis, Italy, 2010 to 2015

**DOI:** 10.2807/1560-7917.ES.2018.23.50.1800032

**Published:** 2018-12-13

**Authors:** Maria Luisa Ricci, Antonella Grottola, Giulia Fregni Serpini, Antonino Bella, Maria Cristina Rota, Francesca Frascaro, Emanuela Pegoraro, Marisa Meacci, Anna Fabio, Elena Vecchi, Antonietta Girolamo, Fabio Rumpianesi, Monica Pecorari, Maria Scaturro

**Affiliations:** 1 Department of Infectious Diseases, Istituto Superiore di Sanità, Rome, Italy; 2 Unit of Microbiology and Virology, Polyclinic University Hospital, Modena, Italy; 3 U.O.C. of Microbiology and Virology, Azienda Ospedaliero-Universitaria, Verona, Italy; 4 Hospital Hygiene, Polyclinic University Hospital, Modena, Italy; 5 Department of Surgery, Medicine, Dentistry and Morphological Scientists with Transplant Surgery, Oncology and Regenerative Medicine Relevance, University of Modena and Reggio Emilia, Modena, Italy

**Keywords:** Legionella, Real-Time PCR, laboratory diagnosis, urinary antigen test, culture

## Abstract

**Aim:**

To evaluate real-time PCR as a diagnostic method for Legionnaires’ disease (LD). Detection of *Legionella* DNA is among the laboratory criteria of a probable LD case, according to the European Centre for Disease Prevention and Control, although the utility and advantages, as compared to culture, are widely recognised.

**Methods:**

Two independent laboratories, one using an in-house and the other a commercial real-time PCR assay, analysed 354 respiratory samples from 311 patients hospitalised with pneumonia between 2010–15. The real-time PCR reliability was compared with that of culture and urinary antigen tests (UAT). Concordance, specificity, sensitivity and positive and negative predictive values (PPV and NPV, respectively) were calculated.

**Results:**

Overall PCR detected eight additional LD cases, six of which were due to *Legionella pneumophila* (Lp) non-serogroup 1. The two real-time PCR assays were concordant in 99.4% of the samples. Considering in-house real-time PCR as the reference method, specificity of culture and UAT was 100% and 97.9% (95% CI: 96.2–99.6), while the sensitivity was 63.6% (95%CI: 58.6–68.6) and 77.8% (95% CI: 72.9–82.7). PPV and NPV for culture were 100% and 93.7% (95% CI: 91.2-96.3). PPV and NPV for UAT were 87.5% (95% CI: 83.6-91.4) and 95.8% (95% CI: 93.5-98.2).

**Conclusion:**

Regardless of the real-time PCR assay used, it was possible to diagnose LD cases with higher sensitivity than using culture or UAT. These data encourage the adoption of PCR as routine laboratory testing to diagnose LD and such methods should be eligible to define a confirmed LD case.

## Introduction

Legionnaires’ disease (LD) is a severe form of pneumonia and is caused by bacteria belonging to the *Legionella* genus. These microorganisms are ubiquitous in natural freshwater environments and can also thrive in man-made water systems. *Legionella pneumophila* (Lp) is the mostly responsible for the development of LD; serogroup 1 (sg1) is most frequently isolated from clinical samples [1]. LD cannot be clinically or radiologically distinguished from pneumonia cases of different aetiology, therefore the disease often remains undiagnosed. Age, underlying diseases, delay in diagnosis and inappropriate antibiotic therapy can result in an increase of the case fatality rate from LD [2].

In 2015, the enumeration of all cases with a known outcome demonstrated an average case fatality rate of 8%, with a higher rate (28%) in nosocomial cases in Europe [3]. According to LD case definition [4,5], culture, a fourfold raise in Lp sg1 antibodies and urinary antigen test (UAT) are the only laboratory methods considered reliable for LD case confirmation. While serology has been nearly abandoned, UAT has almost completely replaced culture, representing 82% and 97% of diagnosis in Europe and in the United States (US), respectively [1,3]. A similar trend was observed in Italy, where in 2016 UAT and culture were used to diagnose 95.5% and 2.7% of cases, respectively [6]. However, both culture and UAT have some limitations; culture is time consuming and has a sensitivity ranging from < 10–80% [1], UAT can be performed rapidly and with very high specificity for Lp sg1, but sensitivity for non-sg1 antigens is very low. In addition, the sensitivity of UAT has been demonstrated to be lower for non-Lp sg1 MAb 3/1-positive strains [2]. Of note, laboratory diagnosis is often based on a single method, without taking into account the limitations that each diagnostic assay might have [3,7].

Diagnostic tools based on detection of nucleic acids are an option to overcome the limitations observed by both culture and UAT. The numerous PCR assays proposed have shown high sensitivity and specificity, provided fast results and were able to detect a higher number of cases, giving the possibility to improve surveillance and better characterise local LD epidemiology [8-14]. Despite an increase in the proportion of cases diagnosed by PCR being reported in several European countries, the use of PCR is still very limited; presently a positive PCR result only defines a LD probable case [4,5]. Currently, in Italy, only 0.1% of LD cases are diagnosed by PCR [6].

The aim of this retrospective study was to evaluate real-time PCR as rapid diagnostic tool to define a LD case.

## Methods

Respiratory samples were analysed using two different real-time PCR assays, performed in two different laboratories.

### Samples collection

A total of 369 respiratory samples (including sputa, bronchial-alveolar lavages and bronchial aspirates) collected from 326 patients admitted to hospital for any pneumonia between 2010 and 2015 in Italy and were retrospectively analysed for *Legionella pneumophila* DNA detection.

Clinical samples were collected by two hospital laboratories, 74 samples (from 74 patients) from the Laboratory of Microbiology and Virology (University Hospital of Verona) and 295 (from 252 patients) from the Modena Regional Reference Laboratory (RRL) for Clinical Diagnosis of Legionellosis (Unit of Microbiology and Virology-Polyclinic University Hospital). All clinical samples were obtained 1 or 2 days after the onset of symptoms except three samples that were collected 5 days after onset of the disease. After collection, respiratory samples were stored at - 80 °C until tested.

Furthermore, 278 urine samples were available from 246 patients. There were 74 urine samples from 74 patients from Verona and 204 urine samples from 172 patients from Modena RRL.

### Culture examination and urinary antigen test

While patients were hospitalised with pneumonia symptoms, the Laboratory of Microbiology and Virology of Verona and the Modena RRL performed *Legionella* culture and UAT. For 25 patients culture was performed on two different respiratory samples and for nine patients on three samples, while for the remaining patients culture was performed on only one sample. Culture was carried out according to the procedures described elsewhere [15].

Both laboratories performed UAT by using both BinaxNOW Legionella Urinary Antigen Card kit and Binax Legionella Urinary Antigen EIA kit (Alere, Scarborough, US). Urine samples were always boiled before testing. For 19 patients UAT was performed on two urinary samples and for eight patients on three samples.

### Real-time PCR

DNA extraction was performed at the Modena RRL using the ELITe STAR 200 Extraction kit (ELITechGroup S.p.A, Torino, Italy). DNA extracts were split in two aliquots to be analysed by real-time PCR at the Modena RRL and at the National Reference Laboratory at the Istituto Superiore di Sanità in Rome.

The Modena RRL analysed 5μL of DNA with the CE IVD marked real-time PCR commercial kit Legionella pn. Q-PCR Alert (ELITechGroup, CE IVD marked) detecting for Lp *mip* gene, according to the manufacturer’s instructions on a 7300 Real-Time PCR System (Applied Biosystems, Foster City, California (CA), US). The NRL also analysed 5μL of DNA using an in-house real-time PCR assay in a final volume of 20μL, containing 10μL of EXPRESS qPCR SuperMix, (Invitrogen, Carlsbad, CA, US), with Chromo 4 BioRad instrument (Bio-Rad, Hercules, CA, US), updated to CFX-96, and the following thermal protocol: 5 minutes at 95°C followed by 45 cycles of denaturation at 95°C for 10 seconds and annealing/extension at 60 °C for 15 seconds. Primers and probes were as described by Mentasti et al. [14], targeting *mip* and *wzm* genes for detection of Lp (sg1–15) and sg1 marker, respectively. Primers and probes for internal control DNA were also as already described [14].

### Statistical analysis

The concordance between tests was evaluated using the Kappa test (K < 0.20 = “poor”; 0.20–0.40 = “fair”; 0.40–0.60 = “moderate”; 0.60–0.80 = “good”; 0.80–1.00 = “very good”). The specificity, sensitivity and the positive and negative predictive values (PPV and NPV, respectively) and 95% confidence intervals (CI) for all methods were calculated considering the in-house real-time PCR as a reference method. In addition, the concordance between all methods was also calculated. All statistical analyses were performed by Stata software version 11.2 (StataCorp, Texas, US).

## Results

### Samples analysed by real-time PCR, culture and urinary antigen test

Of 369 DNA samples, 15 were excluded from the comparison with culture and UAT because they were inhibitory in both PCR assays, as demonstrated by the absence of amplification of the internal control. These samples were also found negative for culture and UAT. Therefore, 354 samples from 311 patients were included in the comparison of PCR results with culture and/or UAT results (Table 1).

**TABLE 1:** Clinical samples analysed for admitted patients, Italy, 2010–15 (n = 311)

	Number of tested samples	Number of positive samples	Number of negative samples	Number of individuals tested
In-house real-time PCR assay	354	55	299	311
Commercial real-time PCR assay	354	53	301	311
UAT	278	40	238	246
Culture	354	35	319	311

PCR: polymerase chain reaction; UAT: urinary antigen test.

Both commercial and in-house real-time PCR assays gave the same results in 352 out of 354 samples, of which 299 (85%) were negative and 53 (15%) were positive (53 positive for *mip* marker and six positive also for *wzm* target). The in-house PCR detected two more positive samples (n = 55) compared with the commercial one. Of the 354 samples analysed by in-house PCR, six samples, (five negatives for both culture and UAT and one negative only for UAT but positive for culture) were identified as Lp non-sg1. Since the in-house PCR assay was able to differentiate Lp sg1 from the other serogroups, it was arbitrarily considered as a reference assay.

The concordance of the two PCR assays (commercial vs in-house) was 99.4% with a K = 0.98 (p < 0.0001). Specificity and sensitivity of commercial PCR assay were calculated equal to 100% and 96.4% (95% CI: 94.4–98.3) respectively.

All 354 respiratory samples were also tested by culture; of these, 35 (9.9%) were positive.

A total of 278 urine samples were tested by UAT and 40 (14.3%) were found positive. The two methods used to detect the urinary antigen were concordant on all tested samples.

### Legionnaires’ disease cases detected

The total number of LD cases detected was 52 (Table 2) and it was calculated considering the patients with at least one positive diagnostic test (culture, UAT and PCR). The in-house PCR assay was considered as a reference for comparison with culture and UAT results.

**Table 2:** Legionnaires’ disease cases with at least one positive diagnostic test, Italy, 2010–15 (n = 52)

Culture	Urinary antigen test	In-house real-time PCR	Number of cases N = 52
Positive	Positive	Positive	21
Positive	Negative	Positive	3
Positive	ND	Positive	5
Negative	Positive	Positive	10
Negative	ND	Positive	3
Negative	Negative	Positive	5
Negative	Positive	Positive	5

ND: not done.

Using culture and/or urinary antigen test for diagnosis, the number of LD cases detected was 44; when the in-house PCR assay was added, the number of detected cases increased to 52 (Table 2). PCR confirmed LD diagnosis in 84.6% of cases with at least one traditional diagnostic criterion positive (culture or UAT or both) and an increment of 18.2% was observed.

The comparison between culture and the in-house real-time PCR assay showed that the sensitivity of culture (63.6%; 95% CI: 58.6–68.6) was lower, while the specificity was 100%. The PPV and the NPV were 100% and 93.7% (95% CI: 91.2-96.3), respectively. Overall concordance was good (94.3%; k = 0.75; p < 0.0001) (Table 3).

**Table 3:** Comparison of culture and UAT vs in-house real-time PCR by sensitivity, specificity, PPV, NPV, concordance and kappa value, Italy, 2010–15

Comparison	Sensitivity (95% CI)	Specificity (95% CI)	PPV (95% CI)	NPV (95% CI)	Concordance (%)	Kappa value	p-value
Culture vs real-time PCR in-house	63.6 (58.6–68.6)	100.0	100.0	93.7 (91.2–96.3)	94.3	0.75	< 0.0001
UAT vs real-time PCR in-house	77.8 (72.9–82.7)	97.9 (96.2–99.6)	87.5 (83.6–91.4)	95.8 (93.5–98.2)	94.6	0.79	< 0.0001

CI: confidence interval; NPV: negative predictive value; PCR: polymerase chain reaction; PPV: positive predictive value; UAT: urinary antigen test.

The comparison between UAT and the in-house PCR showed a higher sensitivity (77.8%; 95% CI: 72.9–82.7) than between culture and PCR, while specificity was slightly lower (97.9%; 95% CI: 96.2–99.6), and PPV and NPV were 87.5% (95% CI: 83.6–91.4) and 95.8% (95% CI: 93.5–98.2) respectively. Overall concordance of the two assays was good (94.6%; k = 0.79; p < 0.0001) (Table 3).

## Discussion

In this study two independent laboratories, using a different real-time PCR assay for *Legionella pneumophila* DNA detection, analysed 354 respiratory samples and provided results with a very high concordance (99.4%).

Our results highlight a higher sensitivity of PCR compared with culture and a higher diagnostic efficiency compared with UAT. Furthermore, as recently stressed by other authors [16,17], it is important to perform more than one diagnostic assay in order to properly diagnose LD. Five of the eight LD cases with negative UAT results would have been missed if PCR assays, able to detect all Lp serogroups, had not been performed. Although in some instances UAT can incidentally detect non-1 Lp serogroups, they are designed to specifically detect Lp1 antigen, therefore, negative UAT results do not completely rule out LD infection. In addition to the aforementioned five cases (negative for UAT and for culture), three more culture-negative cases, resulted positive for Lp DNA by PCR. For these three, clinicians had only requested cultures and did not request UAT. Overall the eight additional cases show that even with a negative diagnosis but in presence of pneumonia, LD infection should be suspected and all available tests performed to investigate it.

Considering that urine samples were boiled before testing to destroy heat-sensitive proteins that could affect the test, false positive results can be reasonably excluded [7]. A possible explanation for the five UAT-positive but PCR-negative cases was obtained querying patients’ records: for two patients a sputum sample was promptly collected and analysed, while for the others sputum analysis was requested 5 or more days after the antibiotic therapy was started. Although there are not sufficient data to show if and how PCR results might be affected by an on-going antibiotic therapy, the above observation suggests the need to perform PCR assay as soon as possible, ideally before or immediately after the initiation of the antibiotic treatment.

The NPV was suggestive of the excellent reliability of the PCR methods, even though only Lp DNA was targeted. However, this limitation can often be found also using culture method, because specific and selective *Legionella* isolation media, such as buffered charcoal yeast extract (BCYE) and glycine vancomycin polymyxin cycloheximide (GVPC), poorly support *Legionella* non-*pneumophila* growth [18]. The PPV was also consistent with a higher sensitivity of PCR than culture.

The reliability of PCR in diagnosing LD is more and more recognised by the scientific community and recent studies demonstrated a better performance of PCR compared with other diagnostic assays, regardless of the type of respiratory sample (bronchoalveolar lavage or sputum) [9,13]. Moreover, PCR can also detect the presence of all *Legionella* species some of which are notoriously difficult to isolate by culture [19].

In this study, the use of real-time PCR resulted in an increment of eight (18.2%) identified LD cases and therefore is an objective improvement in the diagnosis of LD. Real-time PCR has been considered a poorly reliable method due to the risk of cross-contaminations, however, the introduction of automated procedures for DNA extractions and also for PCR set up, has resulted in a consistent improvement in preventing this PCR drawback. Therefore, after an appropriate validation of their own molecular tests, clinical microbiology laboratories can adopt PCR assays to detect *Legionella* in respiratory samples.

The adoption of rapid methods to quickly identify LD cases is a priority, as the infection rate is underestimated all over the world and difficult to quantify, and increasing in several countries [2,3,20]. The laboratory procedures currently used to define confirmed LD cases are not able to guarantee a high level of sensitivity and specificity of results and they can be time-consuming. As a rapid LD diagnosis is crucial for both patient management and public health purposes, real-time PCR should be considered and implemented both locally and at *Legionella* reference laboratories in combination with all the other available methods.

In conclusion, as already observed in other countries, this study shows that the introduction of real-time PCR can improve LD diagnosis and should be considered among the criteria to define confirmed cases of LD [13].

## References

[1] Mercante JW Winchell JM Current and emerging Legionella diagnostics for laboratory and outbreak investigations. Clin Microbiol Rev. 2015;28(1):95-133. 10.1128/CMR.00029-14 25567224 PMC4284297

[2] Phin N Parry-Ford F Harrison T Stagg HR Zhang N Kumar K Epidemiology and clinical management of Legionnaires’ disease. Lancet Infect Dis. 2014;14(10):1011-21. 10.1016/S1473-3099(14)70713-3 24970283

[3] European Centre for Disease Prevention and Control (ECDC). Annual Epidemiological report for 2015. Legionnaires’ disease. Stockholm: ECDC; 2017. Available from: https://ecdc.europa.eu/en/publications-data/legionnaires-disease-annual-epidemiological-report-2015

[4] European Centre for Disease Prevention and Control (ECDC). European Union (EU) case definitions. Stockholm: ECDC; 2018. Available from: https://ecdc.europa.eu/en/infectious-diseases-public-health/surveillance-and-disease-data/eu-case-definitions

[5] Centers for Disease Control and Prevention (CDC). 2012 National Notifiable Infectious Diseases (Historical). Atlanta: CDC; 2012. Available from: https://wwwn.cdc.gov/nndss/conditions/notifiable/2012/infectious-diseases/

[6] L’Istituto Superiore di Sanità (ISS). La legionellosi in Italia nel 2017. [Legionellosis in Italy in 2017]. Rome: ISS; 2018. Italian. Available from: http://old.iss.it/binary/publ/cont/ONLINE.pdf

[7] Rota MC Fontana S Montaño-Remacha C Scaturro M Caporali MG Vullo V Legionnaires’ disease pseudoepidemic due to falsely positive urine antigen test results. J Clin Microbiol. 2014;52(6):2279-80. 10.1128/JCM.00493-14 24719437 PMC4042736

[8] Avni T Bieber A Green H Steinmetz T Leibovici L Paul M Diagnostic Accuracy of PCR Alone and Compared to Urinary Antigen Testing for Detection of Legionella spp.: a Systematic Review. J Clin Microbiol. 2016;54(2):401-11. 10.1128/JCM.02675-15 26659202 PMC4733173

[9] Botelho-Nevers E Grattard F Viallon A Allegra S Jarraud S Verhoeven P Prospective evaluation of RT-PCR on sputum versus culture, urinary antigens and serology for Legionnaire’s disease diagnosis. J Infect. 2016;73(2):123-8. 10.1016/j.jinf.2016.04.039 27306488

[10] Chen DJ Procop GW Vogel S Yen-Lieberman B Richter SS Utility of PCR, Culture, and Antigen Detection Methods for Diagnosis of Legionellosis. J Clin Microbiol. 2015;53(11):3474-7. 10.1128/JCM.01808-15 26292304 PMC4609676

[11] Benitez AJ Winchell JM Clinical application of a multiplex real-time PCR assay for simultaneous detection of Legionella species, Legionella pneumophila, and Legionella pneumophila serogroup 1. J Clin Microbiol. 2013;51(1):348-51. 10.1128/JCM.02510-12 23135949 PMC3536254

[12] Murdoch DR Diagnosis of Legionella infection. Clin Infect Dis. 2003;36(1):64-9. 10.1086/345529 12491204

[13] Murdoch DR Podmore RG Anderson TP Barratt K Maze MJ French KE Impact of routine systematic polymerase chain reaction testing on case finding for Legionnaires’ disease: a pre-post comparison study. Clin Infect Dis. 2013;57(9):1275-81. 10.1093/cid/cit504 23899682

[14] Mentasti M Kese D Echahidi F Uldum SA Afshar B David S Design and validation of a qPCR assay for accurate detection and initial serogrouping of Legionella pneumophila in clinical specimens by the ESCMID Study Group for Legionella Infections (ESGLI). Eur J Clin Microbiol Infect Dis. 2015;34(7):1387-93. 10.1007/s10096-015-2363-4 25851812

[15] Istituto Superiore di Sanità (ISS). Linee guida per la prevenzione ed il controllo della legionellosi [Guidelines for prevention and control of legionellosis]. Rome: ISS; 2015. Italian. Available from: http://old.iss.it/binary/iss4/cont/C_17_pubblicazioni_2362.pdf

[16] Peci A Winter AL Gubbay JB Evaluation and Comparison of Multiple Test Methods, Including Real-time PCR, for Legionella Detection in Clinical Specimens. Front Public Health. 2016;4:175. 10.3389/fpubh.2016.00175 27630979 PMC5005417

[17] Svarrer CW Lück C Elverdal PL Uldum SA Immunochromatic kits Xpect Legionella and BinaxNOW Legionella for detection of Legionella pneumophila urinary antigen have low sensitivities for the diagnosis of Legionnaires’ disease. J Med Microbiol. 2012;61(2):213-7. 10.1099/jmm.0.035014-0 21921112

[18] Lee JV Lai S Exner M Lenz J Gaia V Casati S An international trial of quantitative PCR for monitoring Legionella in artificial water systems. J Appl Microbiol. 2011;110(4):1032-44. 10.1111/j.1365-2672.2011.04957.x 21276147 PMC3564408

[19] Vaccaro L Izquierdo F Magnet A Hurtado C Salinas MB Gomes TS Correction: First Case of Legionnaire’s Disease Caused by Legionella anisa in Spain and the Limitations on the Diagnosis of Legionella non-pneumophila Infections. PLoS One. 2016;11(9):e0162934. 10.1371/journal.pone.0162934 27442238 PMC4956277

[20] Farnham A Alleyne L Cimini D Balter S Legionnaires’ disease incidence and risk factors, New York, New York, USA, 2002-2011. Emerg Infect Dis. 2014;20(11):1795-802. 10.3201/eid2011.131872 25513657 PMC4214295

